# Case Report: Hematopoietic stem cell transplantation for refractory chronic immune thrombocytopenia in a child

**DOI:** 10.3389/fimmu.2026.1908385

**Published:** 2026-09-04

**Authors:** Fan Liu, Hongjuan Li, Yan Gu, Yan Han, Guoyu Ding, Yuqi Zhao, Xiaoyue Zhang, Xue Li, Huidi Feng, Yixiao Gong, Hongmei Wang

**Affiliations:** Department of Pediatric Hematology, The First Affiliated Hospital of Shandong First Medical University & Shandong Provincial Qianfoshan Hospital, Jinan, Shandong, China

**Keywords:** allogeneic hematopoietic stem cell transplantation, case report, graft-versus-host disease, pediatric, refractory immune thrombocytopenia

## Abstract

Immune thrombocytopenia (ITP) is an autoimmune bleeding disorder characterized by immune-mediated platelet destruction and impaired platelet production. While pediatric ITP is often self-limited or responsive to first-line therapies, such as corticosteroids and intravenous immunoglobulin (IVIG), a small subset develop refractory chronic disease. Hematopoietic stem cell transplantation (HSCT) has been explored as a potential immune-reconstituting rescue intervention for highly selected patients with multiple treatment failures, prolonged severe thrombocytopenia, and persistent bleeding risk. Here, we report a case of a 6-year-old child with refractory chronic ITP who had severe thrombocytopenia and recurrent life-threatening bleeding despite 11 first-line, second-line, and salvage treatment regimens and ultimately underwent 10/10 HLA-matched unrelated donor allogeneic HSCT (allo-HSCT). After transplantation, the patient experienced delayed platelet recovery, acute graft-versus-host disease (aGvHD), viral reactivation and transient transplant-associated thrombotic microangiopathy (TA-TMA). After repeated treatment adjustments guided by close real-time clinical monitoring, he eventually achieved stable trilineage engraftment and has remained in complete remission (platelet count ≥100×10^9^/L) without major bleeding events for more than 11 months. We also review the available literature on HSCT for refractory ITP. This case highlights the therapeutic challenges of pediatric refractory chronic ITP, adds detailed longitudinal clinical evidence to the limited literature on HSCT in this setting, and supports consideration of allo-HSCT as a potential salvage option in exceptionally selected patients.

## 1 Introduction

Immune thrombocytopenia (ITP) is a common acquired bleeding disorder and is often self-limiting in children, with the majority of patients responding to first-line treatments such as corticosteroids and intravenous immunoglobulin (IVIG) (1, 2). Approximately 20%-25% of pediatric ITP cases progress to chronic ITP (cITP), defined as disease lasting more than 12 months (1, 3). Although most children with cITP can be managed with conventional therapies, 4%-20% of patients are difficult to manage and become refractory to standard treatment (4, 5). Refractory ITP presents a major therapeutic challenge. Miltiadous et al. defined it as very low platelet counts with bleeding, failure of at least two treatments, and no single medication providing reliable control, without necessarily requiring prior splenectomy (4, 6). The North American Pediatric ITP Consortium identified as a particularly challenging subgroup children with persistent platelet nonresponse or a high disease burden despite disease-modifying therapies targeting at least two distinct mechanisms, including thrombopoietin receptor agonists (TPO-RAs), rituximab, immunomodulatory agents, and splenectomy (7). Apparent refractoriness may reflect either true treatment-resistant primary ITP or diagnostic misclassification, including secondary ITP and alternative diagnoses such as inherited thrombocytopenia, bone marrow failure syndromes, myelodysplastic syndromes, and drug-induced thrombocytopenia (4, 8). Therefore, repeated diagnostic reassessment is essential before further therapeutic escalation, particularly in patients with prolonged severe thrombocytopenia and poor response to multiple therapies. Refractory ITP carries greater disease burden, including lower platelet counts, more frequent bleeding, increased risk of intracranial hemorrhage, and a higher need for treatment, underscoring the need for effective interventions (6, 9).

Hematopoietic stem cell transplantation (HSCT) has been considered as a potential salvage strategy in exceptionally severe cases of refractory ITP when conventional therapies fail. The rationale for HSCT in refractory autoimmune disease is immune ablation followed by immune reconstitution, with the goal of eliminating autoreactive immune clones and re-establishing immune tolerance (10). Both autologous and allogeneic HSCT have been explored in refractory ITP. Autologous HSCT (auto-HSCT) avoids donor-related complications and graft-versus-host disease (GvHD), whereas allogeneic HSCT (allo-HSCT) may offer more profound immune replacement and a potential graft-versus-autoimmunity effect, but with greater treatment-related risks (11, 12). HSCT is not widely used in ITP because of substantial treatment-related morbidity and mortality, and the available evidence remains limited to case reports, registry summaries, and mixed cohorts of autoimmune cytopenias in which ITP is often analyzed together with Evans syndrome, autoimmune hemolytic anemia, or autoimmune neutropenia. As a result, patient selection, transplant platform, conditioning intensity, and peri-transplant management remain poorly defined in ITP, highlighting the need for further disease-specific evidence.

Here, we report a child with exceptionally refractory chronic ITP who ultimately underwent matched unrelated donor (MUD) allo-HSCT after failure of multiple conventional and salvage treatments. This case illustrates the clinical rationale for proceeding to transplantation, the key considerations in treatment decision-making, the challenges of the post-transplant course and the outcome. We also reviewed the published literature on HSCT as a treatment for ITP.

## 2 Case description

### 2.1 Patient information

**Figure 1:**
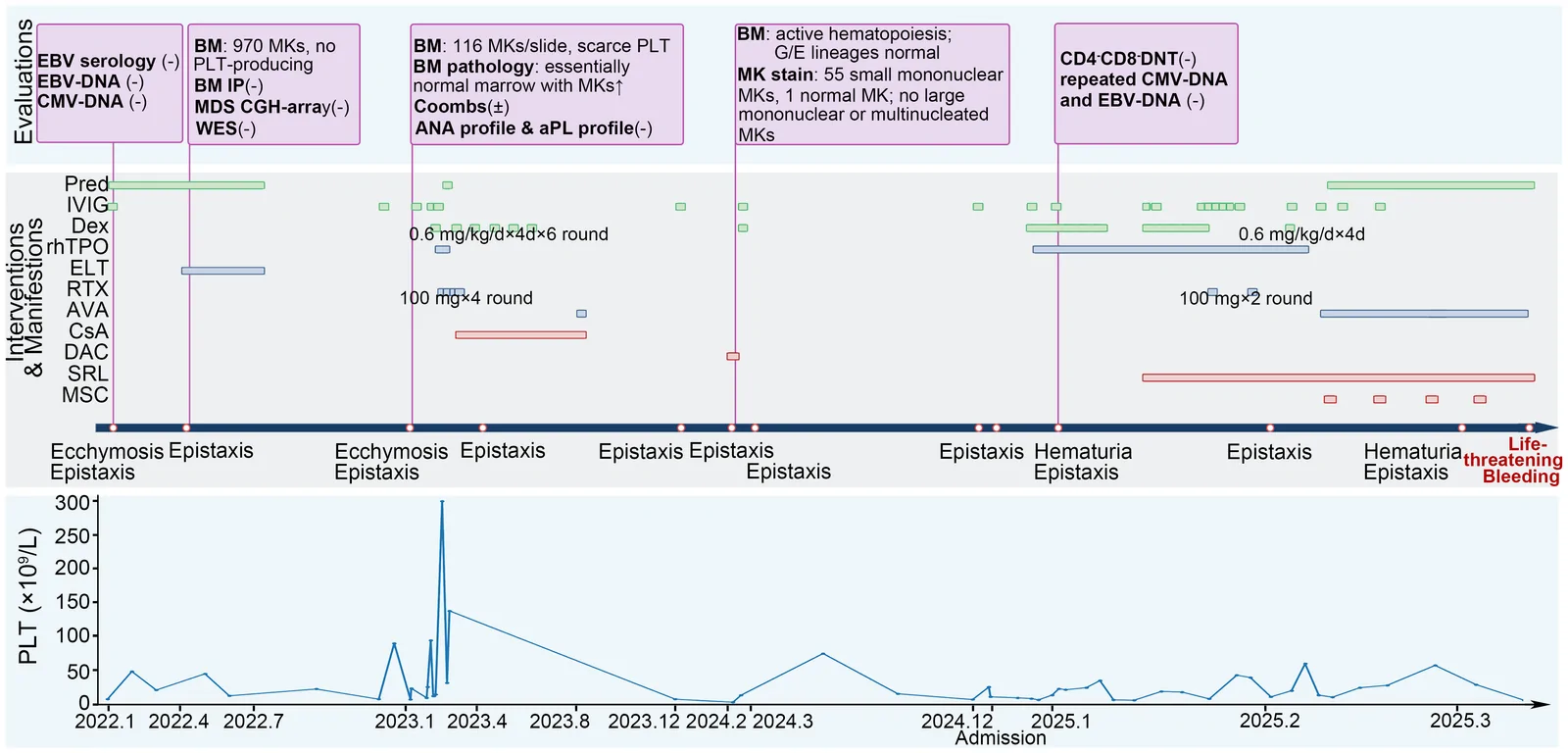
Summary of treatment before allo-HSCT. The upper panel summarizes major diagnostic evaluations and therapeutic interventions. Colored bars in the middle panel represent the timing and duration of individual treatments. The lower panel shows serial platelet counts over time. UC-MSCs were administered at 2.0 × 10^6^ cells/kg per infusion once weekly for four consecutive weeks. ANA, antinuclear antibody; aPL, antiphospholipid antibody; AVA, avatrombopag; BM, bone marrow; CGH-array, comparative genomic hybridization array; CsA, cyclosporine A; DAC, decitabine; Dex, dexamethasone; DNT, double-negative T-cell assay; ELT, eltrombopag; IP, immunophenotyping; IVIG, intravenous immunoglobulin; MDS, myelodysplastic syndromes; MK, megakaryocyte; UC-MSC, umbilical cord-derived mesenchymal stromal cell; PLT, platelet; Pred, prednisone; rhTPO, recombinant human thrombopoietin; RTX, rituximab; SRL, sirolimus; WES, whole-exome sequencing.

A 6-year-old boy initially presented in January 2022 with ecchymosis and epistaxis. Complete blood count showed isolated severe thrombocytopenia, with a platelet count of 5×10^9^/L. Bone marrow aspiration revealed active trilineage hematopoiesis with predominant increased megakaryocytes and reduced platelet-producing forms, supporting the diagnosis of ITP. Initial treatment with IVIG and prednisone resulted in a transient response, but platelet counts declined again shortly thereafter. In April 2022, recurrent epistaxis prompted treatment with prednisone and eltrombopag. However, platelet counts could not be maintained during prednisone tapering, and alternative therapies, including traditional Chinese medicine, provided only transient improvement. In January 2023, he developed recurrent mucocutaneous bleeding and was re-evaluated for refractory ITP. Over the next several months, he underwent combined treatment regimens, including recombinant human thrombopoietin (rhTPO), repeated high-dose dexamethasone, rituximab, cyclosporine (CsA), and avatrombopag, none of which resulted in sustained platelet recovery. In December 2023, his platelet count dropped to 5×10^9^/L, and he received platelet transfusion support together with IVIG. From February 2024 onward, further therapies, including decitabine, IVIG, dexamethasone, and traditional Chinese medicine (herbal regimen details from the outside hospital were unavailable), also failed to provide durable benefit. The prolonged disease course caused recurrent hospitalization, hemorrhagic anemia, and marked limitation of school attendance and daily activity (**Figure 1**).

### 2.2 Clinical findings and diagnostic assessment

The patient was first admitted to our center in December 2024 for recurrent severe thrombocytopenia and 3 days of epistaxis. At referral, the most important findings were recurrent mucocutaneous bleeding, including difficult-to-control epistaxis, ecchymoses, and hematuria, complicated by hemorrhagic anemia. There was no splenomegaly, hypersplenism, lymphadenopathy, or clinical evidence of another systemic disorder explaining thrombocytopenia. Rescue treatments produced only transient platelet increments.

Comprehensive reassessment, including EBV and CMV testing (**Supplementary Table 1**), bone marrow smear and pathology, bone marrow immunophenotyping, direct antiglobulin testing, autoantibody screening, antiphospholipid antibody testing, thyroid function testing, double-negative T-cell analysis, chromosomal microarray, and whole-exome sequencing (WES), did not identify secondary thrombocytopenia or an alternative inherited cause of thrombocytopenia (**Figure 1**).

Given the long-standing platelet count of ≤10×10^9^/L, recurrent bleeding manifestations and failure of multiple treatments before referral to our center, the patient was diagnosed with refractory chronic ITP. After admission, an individualized combination strategy was implemented, including IVIG, corticosteroids, rhTPO, avatrombopag, and rituximab. Sirolimus and umbilical cord-derived mesenchymal stromal cell (UC-MSC) infusion were also used as exploratory interventions for refractory ITP (6, 13, 14). Platelets temporarily increased to 105×10^9^/L but could not be maintained. Other TPO-RAs were not used: romiplostim remained off-label for pediatric ITP in China and was not available, hetrombopag also remained off-label with less published evidence than avatrombopag at that time. In March 2025, the patient developed severe epistaxis and hematuria, with platelets falling to 2×10^9^/L (**Figure 1**).

The role of splenectomy was carefully evaluated. High-dose dexamethasone could transiently increase the platelet count, providing an opportunity to assess splenectomy, but the effect was not sustained, with platelet levels rapidly returning to single digits during steroid tapering and severe epistaxis recurring, requiring red blood cell transfusion. Given this unstable clinical course and the persistent high bleeding risk, repeated multidisciplinary discussion, including pediatric surgical consultation, considered the perioperative bleeding risk to be high and the safe platelet levels could not be reliably maintained. Therefore, splenectomy was deferred.

### 2.3 Transition to allo-HSCT

**Figure 2:**
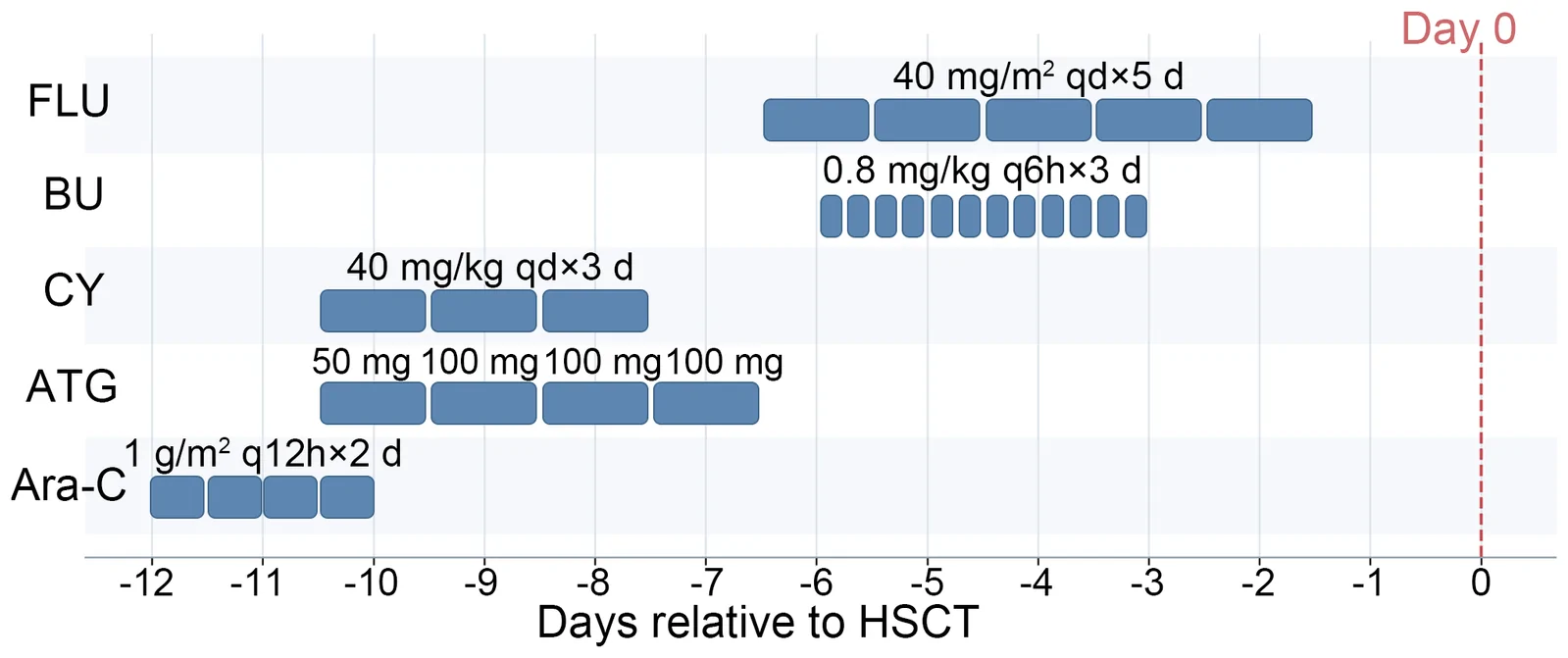
Conditioning timeline before unrelated donor allo-HSCT. Timeline of the myeloablative conditioning regimen relative to allo-HSCT in this patient. Day 0 indicates stem cell infusion. The conditioning regimen consisted of Ara-C at 1 g/m^2^ every 12 hours for 2 days, CY at 40 mg/kg once daily for 3 days, ATG at a total dose of 8.5 mg/kg administered over 4 days, FLU at 40 mg/m^2^ once daily for 5 days, and BU at 0.8 mg/kg every 6 hours for 3 days. Bars indicate the timing and duration of each agent before transplantation. Ara-C, cytosine arabinoside; ATG, antithymocyte globulin; BU, busulfan; CY, cyclophosphamide; FLU, fludarabine.

Given the persistent severe thrombocytopenia with recurrent mucocutaneous bleeding, progression to life-threatening hemorrhagic events, failure of 11 standard and salvage treatment regimens, prolonged dependence on inpatient care, as well as substantial impairment of the patient’s and family’s quality of life, allo-HSCT was ultimately considered as an individualized salvage intervention after multidisciplinary evaluation. The potential benefits of durable disease control and relief from bleeding- and treatment-related burden were carefully balanced against anticipated peri-transplant risks and long-term complications. Following shared decision-making with his mother, transplantation was undertaken. No sibling donor was available, and paternal haploidentical donation was not feasible because the patient’s father was deceased. A healthy HLA 10/10-matched unrelated male donor was identified through the China Marrow Donor Program. Pre-transplant evaluation revealed adrenal insufficiency and subclinical hypothyroidism, which were managed with hydrocortisone and levothyroxine replacement. The patient was admitted to the laminar airflow ward on March 21, 2025, and received myeloablative conditioning with fludarabine (FLU), busulfan (BU), cyclophosphamide (CY), cytosine arabinoside (Ara-C), and antithymocyte globulin (ATG) (**Figure 2**). On April 2, 2025, he underwent peripheral blood stem cell transplantation from the HLA 10/10-matched unrelated donor. The transplant was major ABO-incompatible (donor AB RhD-positive; recipient B RhD-positive), with infused doses of 11.26×10^8^/kg mononuclear cells and 7.62×10^6^/kg CD34^+^ cells.

Anti-infective and immunosuppressive therapies were adjusted dynamically. GvHD prophylaxis consisted of CsA, mycophenolate mofetil (MMF), and short-course methotrexate (MTX). Because of persistently positive fungal biomarkers before conditioning and prolonged immunosuppressive exposure, caspofungin was administered as preemptive antifungal therapy. After transplantation, febrile neutropenia prompted escalation of antibacterial treatment. Acyclovir was administered as routine antiviral prophylaxis. Subsequent viral surveillance, together with clinical manifestations, identified BK virus-associated hemorrhagic cystitis and a rising whole-blood CMV DNA load, which were treated sequentially with ganciclovir, letermovir, and foscarnet. Prophylaxis against veno-occlusive disease/sinusoidal obstruction syndrome (VOD/SOS) consisted of prostaglandin E1 (PGE1) and ursodeoxycholic acid.

**Figure 3:**
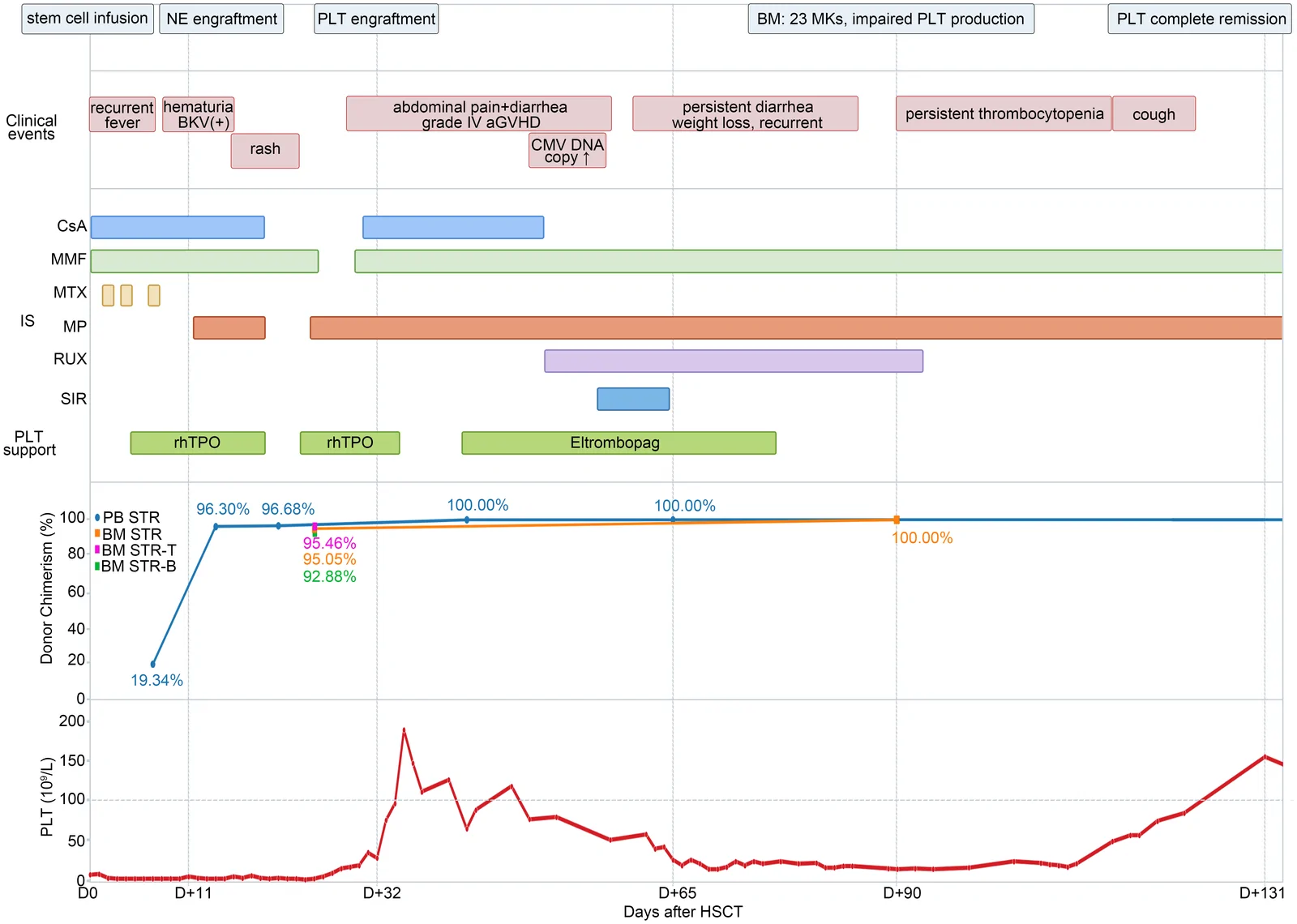
Dynamic immunosuppressive adjustment, donor chimerism, and platelet recovery up to day +131 after HSCT. The upper panel summarizes major clinical events. The middle panel shows adjustment of immunosuppressive and platelet-supportive treatment over time. The lower panels depict serial donor chimerism by STR analysis in peripheral blood and bone marrow, together with longitudinal platelet counts. Peripheral blood donor chimerism increased from 19.34% on day +7 to 96.30% on day +14 and 96.68% on day +21, reached 100% by day +42, and remained complete thereafter. Bone marrow donor chimerism was 95.05% on day +25 and reached 100% on day +90. On day +25, bone marrow donor T-cell and B-cell chimerism were 95.46% and 92.88%, respectively. Longer-term follow-up beyond day +131 is described in the main text. aGvHD, acute graft-versus-host disease; BKV, BK virus; BM, bone marrow; MKs, megakaryocytes; CMV, cytomegalovirus; IS, immunosuppression; CsA, cyclosporine A; MMF, mycophenolate mofetil; MP, methylprednisolone; MTX, methotrexate; NE, neutrophil engraftment; PB, peripheral blood; PLT, platelet; RUX, ruxolitinib; SIR, sirolimus; rhTPO, recombinant human thrombopoietin; STR, short tandem repeat; TA-TMA, transplant-associated thrombotic microangiopathy.

Neutrophil engraftment was achieved on day +11, whereas platelet recovery was delayed, requiring treatment with IVIG and rhTPO. Early post-transplant donor chimerism increased from 19.34% in the first week after transplantation to 96.30% in the second week and 96.68% in the third week, indicating primary graft failure less likely (**Figure 3**). Persistently low platelet counts (1-5×10^9^/L) prompted the tapering and discontinuation of CsA on day +19 and MMF on day +25, concurrent with the initiation of methylprednisolone (1 mg/kg per dose every 12 hours). The platelet count subsequently began to rise gradually, with platelet engraftment achieved on day +32. However, the patient developed a rash accompanied by diarrhea on day +29, prompting the immediate reintroduction of oral MMF and intravenous CsA. Despite this, the diarrhea progressively worsened, leading to a diagnosis of severe aGvHD (grade IV by MAGIC criteria; skin stage 1, lower gastrointestinal stage 4). Because symptoms remained recurrent and steroid tapering was difficult, ruxolitinib and sirolimus were added sequentially, alongside supportive care. During this period, the patient developed a transient episode of transplant-associated thrombotic microangiopathy (TA-TMA), which improved after sirolimus withdrawal. Despite improvement in GvHD, the platelet count again declined and remained below 50×10^9^/L after day +58. Donor chimerism had remained 100% and bone marrow evaluation showed trilineage hematopoiesis with impaired platelet-producing megakaryopoiesis, favoring possible immune-mediated thrombocytopenia. With supportive care, IVIG, TPO-RA therapy and tailored immunosuppressive management, the platelet count gradually increased and remained ≥100×10^9^/L after day +131 (**Figure 3**). At 15 months after HSCT, the patient remained in complete remission. Serial peripheral blood immune cell subset monitoring documented gradual quantitative recovery of T-cell, B-cell, and NK-cell populations over time (**Supplementary Table 2**). During follow-up, he developed mild chronic graft-versus-host disease (cGvHD) with skin and liver involvement, which was managed with ruxolitinib, MMF, and tacrolimus. By the most recent assessment in July 2026, hyperpigmentation in the extremities and back had diminished, liver function tests were largely within normal limits, and no other manifestations of cGvHD were observed. Tacrolimus was being gradually tapered. Thyroid function remained stable on levothyroxine replacement, whereas secondary adrenal insufficiency related to prior prolonged corticosteroid exposure had resolved. He had impaired linear growth before HSCT, likely related to prolonged corticosteroid exposure. His height increased by 5 cm over the preceding 6 months, suggesting recovery toward an age-appropriate growth velocity. He had also returned to school and resumed near-normal age-appropriate daily and social activities.

## 3 Discussion

This case represents refractory chronic ITP with persistent severe thrombocytopenia, recurrent bleeding, poor response to multiple lines of therapy and cumulative treatment-related toxicity. Despite exposure to corticosteroids, IVIG, thrombopoietin-based agents, rituximab, CsA, decitabine, sirolimus, and UC-MSC infusion, our patient achieved only transient platelet responses and developed life-threatening bleeding, which ultimately supported the decision to proceed with MUD allo-HSCT as a salvage immune-reconstructive strategy.

Published literature on HSCT for refractory ITP remains sparse, and most reports involve heterogeneous autoimmune cytopenia cohorts in which ITP-specific outcomes cannot be separately analyzed (**Table 1**). Across these series, patients uniformly represent the multi-line treatment failure before HSCT. Durable remission can be achieved in a subset of patients, but transplant-related morbidity and mortality remain important concerns. For example, in the EBMT series, 2 of 12 patients died, and the CIBMTR report also documented fatal peritransplant intracranial hemorrhage (15, 16). Historical experience has been dominated by auto-HSCT, particularly in earlier reports, and many of those patients had already failed splenectomy before transplantation (11, 17–19). Allo-HSCT has been reported less frequently; however, limited registry data have suggested a trend toward fewer non-responses and relapses compared with auto-HSCT, although this difference was not statistically significant (11, 20). This observation is biologically plausible, as conditioning may deplete the autoreactive host immune repertoire, while donor-derived hematopoiesis may provide a platform for immune reconstitution and potential re-establishment of tolerance (10). No standard conditioning protocol currently exists, and previously reported patients have received heterogeneous regimens, ranging from high-dose CY-based auto-HSCT to reduced-intensity or myeloablative allogeneic regimens. In the present case, a myeloablative conditioning regimen was selected because of the markedly active hematopoiesis and the extremely refractory disease course, with the aim of maximizing eradication of resistant autoreactive immune clones, thoroughly clearing the marrow niche to facilitate donor stem cell engraftment, and minimizing the risk of post-transplant relapse.

**Table 1:** Published reports of HSCT for refractory ITP and mixed autoimmune cytopenia cohorts including ITP.

Ref	Study design	Disease category	No. of ITP	HSCT indication	HSCT type	Conditioning	Main outcome	Follow-up
Skoda et al. (17)	Case report	Refractory cITP	1	Failed steroids, splenectomy, CsA, danazol, protein A immunoadsorption	auto-HSCT	High-dose CY	Transient platelet rise to 42×10^9^/L, then returned to baseline; no sustained response.	6 months
Lim et al. (18)	Case report	Refractory cITP	2	Failed IVIG, steroids, splenectomy; one also received Campath-1H	auto-HSCT	High-dose CY	Both achieved CR.	7–11 months
Huhn et al. (19)	Phase 1/2 (NIH)	Refractory chronic AITP	14	Failed steroids, splenectomy, IVIG, and multiple immunomodulatory therapies	auto-HSCT	High-dose CY	6 durable CR, 2 durable PR, 6 no response.	9–42 months
Passweg et al. (15)	Registry (EBMT)	Autoimmune cytopenias including ITP	12	Failed multiple treatments	auto-HSCT	Heterogeneous	2 deaths, 3 NR, 3 transient responses, 4 sustained remissions, 1 not evaluated.	3–84 months (median 43)
Passweg et al. (11)	Registry update (EBMT)	Refractory autoimmune cytopenias including ITP	20	Long-standing disease after multiple prior treatments	auto-HSCT (17); allo-HSCT (3)	Heterogeneous	ITP outcomes not separately reported. Responses in a substantial proportion; possibly higher rates after allo-HSCT.	Not separately reported
Rabusin et al. (20)	Retrospective registry (pediatric)	Treatment-resistant autoimmune cytopenias including ITP	4	Failed numerous lines of immunosuppressive therapy	auto-HSCT	Heterogeneous	3 ITP patients achieved CR; 1 died. Relapse/failure tended to be more frequent after auto-HSCT (not significant).	113–132 months (median 126)
Pasquini et al. (16)	Multicenter registry (CIBMTR)	AID including autoimmune cytopenias	Not separately reported	Severe AID after failure of other therapies	auto-HSCT and allo-HSCT	Heterogeneous	2 deaths from peritransplant intracranial hemorrhage (auto group); others survived.	31 months (auto); 24 months (allo)
Vaughn et al. (10)	Narrative review	Refractory ITP and Evans syndrome	N/A	Refractory/intolerant to multiple prior therapies	auto-HSCT and allo-HSCT	Heterogeneous	HSCT may provide definitive therapy in selected patients; evidence base limited; patient selection crucial.	N/A

HSCT, hematopoietic stem cell transplantation; cITP, chronic immune thrombocytopenia; CsA, cyclosporine A; auto-HSCT, autologous HSCT; allo-HSCT, allogeneic HSCT; CY, cyclophosphamide; IVIG, intravenous immunoglobulin; CR, complete remission; AITP, autoimmune thrombocytopenia; PR, partial remission; NR, no response; EBMT, European Society for Blood and Marrow Transplantation; AID, autoimmune disease; CIBMTR, Center for International Blood and Marrow Transplant Research; N/A, not applicable.

Given the extreme scarcity of detailed, disease-specific reports at this level of treatment refractoriness, the particular value of this case lies in providing a comprehensive longitudinal account—from sequential treatment failure and transplant decision-making through the full post-transplant course—of allo-HSCT for pure refractory ITP in a child. In this case, WES did not identify pathogenic variants associated with known inborn errors of immunity and inherited bone marrow failure syndromes. Together with the comprehensive clinical and laboratory reassessment, these findings did not reveal an alternative inherited diagnosis. Against this diagnostic background, the clinical course was defined by failure of multiple treatment lines, persistent severe thrombocytopenia, recurrent life-threatening bleeding and exceptionally high disease burden. Moreover, prolonged exposure to corticosteroids, immunosuppressive agents, and rituximab had caused profound B-cell depletion, recurrent infections, and adrenal insufficiency. Splenectomy remained an established option, but it was not pursued after multidisciplinary assessment because safe perioperative platelet levels could not be reliably maintained. After multiple standard and salvage medical therapies had failed, the balance between ongoing disease- and treatment-related morbidity and the anticipated risks of transplantation supported consideration of HSCT as an individualized salvage immune-reconstructive strategy.

Importantly, the anticipated benefit of durable hematologic remission and bleeding control had to be carefully weighed against both peri-transplant and long-term transplantation-related risks. These included conditioning-related toxicity, severe infection, graft failure, aGvHD, and transplant-associated thrombotic microangiopathy, as well as potential late complications such as cGvHD, impaired fertility or gonadal dysfunction, endocrine abnormalities, growth impairment, delayed immune reconstitution, and subsequent malignant neoplasms. During follow-up, the patient developed mild cGvHD involving the skin and liver. Following treatment with ruxolitinib, MMF, and tacrolimus, his cutaneous manifestations improved, liver biochemical abnormalities were largely controlled, and immunosuppressive therapy was being gradually tapered. Thyroid function was stable on replacement therapy, secondary adrenal insufficiency resolved, and linear growth was recovering. Long-term surveillance will continue to include assessment for relapse, cGvHD and immune recovery; serial monitoring of height, weight, body mass index, pubertal development, bone health, thyroid function, gonadal function, late organ dysfunction, as well as age-appropriate fertility counseling and semen analysis when appropriate (21). Monitoring for subsequent malignant neoplasms will include routine clinical follow-up, regular complete blood counts, risk-adapted skin and oral cavity, thyroid, and lymph nodes (21).

After transplantation, immunosuppression had to be carefully balanced against graft status--a challenge perfectly illustrated by the clinical dilemma of delayed platelet recovery. Because neutrophil engraftment was achieved early and donor chimerism increased steadily (reaching 96.68% by the third week), persistent severe thrombocytopenia was less suggestive of primary graft failure, but more likely driven by an immune-mediated component and the suppressive effects of concurrent immunosuppressive therapy. This clinical reasoning prompted the proactive tapering and discontinuation of CsA and MMF. While this bold adjustment successfully facilitated initial platelet engraftment, the rapid reduction in immunosuppression precipitated severe aGvHD. This, in turn, forced a difficult trade-off, requiring the prompt reintroduction of CsA and MMF, alongside systemic corticosteroids and the sequential addition of ruxolitinib and sirolimus. Thus, the favorable outcome reflects meticulous and dynamic peri-transplant management, especially the delicate balance between graft-versus-host immune reactions and hematologic recovery.

## 4 Limitations

Several limitations should be considered when interpreting this case. First, allo-HSCT was performed without prior splenectomy. Although splenectomy was repeatedly considered, it was not pursued after an individualized appraisal of the anticipated perioperative risks and potential benefits. Consequently, this case should not be compared directly with cohorts where splenectomy had already been performed. Moreover, this case should not be interpreted as support for bypassing splenectomy. Second, TPO-RA therapy was not exhaustively optimized before allo-HSCT. Romiplostim and hetrombopag were not administered. Switching TPO-RAs may induce responses in some patients with an inadequate response to the initial TPO-RA (22). Third, follow-up remains relatively short. Continued surveillance is needed to confirm durable remission and detect late allo-HSCT complications. Finally, as a single-case report, this experience cannot define the optimal indications, timing, efficacy, or risk-benefit profile of allo-HSCT for refractory pediatric ITP. Collaborative multicenter studies with prolonged follow-up will be required to better address these questions.

## 5 Conclusion

This case suggests that allo-HSCT may induce sustained remission in selected children with life-threatening refractory ITP after failure of multiple conventional and salvage therapies. The benefit of transplantation in this child was reflected not only in hematologic remission but also in quality of life, as he was ultimately able to return to school and near-normal daily life after a prolonged course marked by recurrent severe epistaxis, repeated hospitalizations, intensive immunosuppression, and steroid-related toxicity. The patient has maintained platelet complete remission for more than 11 months, while longer follow-up is still needed to evaluate the durability of response, the risk of cGvHD, and the possibility of late relapse. Further multicenter data are also needed to better define optimal indications and transplant strategies.

## 6 Patient perspective

From the onset of illness, recurrent severe bleeding episodes—including persistent epistaxis, ecchymoses, and hematuria—necessitated frequent emergency hospitalizations throughout the subsequent 3-year treatment course, progressively disrupting the patient’s school attendance and social development during a formative period of childhood. His mother described living with constant fear that another episode of uncontrolled bleeding might occur at any time. As the patient’s sole caregiver after the death of his father, she experienced substantial emotional and practical burden during the prolonged course of treatment failure.

After admission to our center, the patient’s mother strongly wished to proceed with HSCT. The clinical team acknowledged her perspective while explaining that HSCT is not a standard treatment for ITP, representing an exceptional salvage option reserved for demonstrable multi-line treatment failure. The team therefore continued to pursue combination strategies and novel therapeutic approaches. However, after repeated failure of available therapies and progression to life-threatening bleeding, allo-HSCT was undertaken as the individualized salvage intervention.

Following transplantation and stable hematological remission, the patient experienced substantial improvement in his overall well-being. The cessation of bleeding and progressive withdrawal of intensive immunosuppression alleviated the persistent fear that had pervaded daily life. He returned to school and resumed age-appropriate social activities. His mother reported that his clinical recovery had transformed their family life, restoring security, autonomy, and hope for the future.

## Data Availability

The raw data supporting the conclusions of this article will be made available by the authors upon reasonable request, without undue reservation.
