# Supplementary material for: Case Report: Hematopoietic stem cell transplantation for refractory chronic immune thrombocytopenia in a child
Source: Front Immunol. 2026 Sep 4;17:1908385. doi: 10.3389/fimmu.2026.1908385 (PMC13585649; doi:10.3389/fimmu.2026.1908385)
Supplement: Supplementary file 1 [file Table1.docx]

Table S1. Serial EBV-DNA and CMV-DNA monitoring after admission.

| Date | EBV-DNA | CMV-DNA |
| --- | --- | --- |
| 2025-01-13 | Below the lower limit of detection | Not tested |
| 2025-01-27 | Below the lower limit of detection | Below the lower limit of detection |
| 2025-02-05 | Below the lower limit of detection | Not tested |
| 2025-03-12 | Below the lower limit of detection | Below the lower limit of detection |
| 2025-03-24 | Below the lower limit of detection | Below the lower limit of detection |
| 2025-03-27 | Below the lower limit of detection | Below the lower limit of detection |
| 2025-03-31 | Below the lower limit of detection | Below the lower limit of detection |
| 2025-04-03 | Below the lower limit of detection | Below the lower limit of detection |
