# Supplementary material for: Case Report: Hematopoietic stem cell transplantation for refractory chronic immune thrombocytopenia in a child
Source: Front Immunol. 2026 Sep 4;17:1908385. doi: 10.3389/fimmu.2026.1908385 (PMC13585649; doi:10.3389/fimmu.2026.1908385)
Supplement: Supplementary file 2 [file Table2.docx]

Table S2. Serial peripheral blood immune cell subset counts before and after allo-HSCT.

| Time point relative  to HSCT | B cells (/μL) | CD4+ T cells (/μL) | CD8+ T cells (/μL) | NK cells (/μL) |
| --- | --- | --- | --- | --- |
| Day -21 | 0 | 424 | 556 | 9 |
| Day +42 | 4 | 116 | 214 | 46 |
| Day +62 | 10 | 100 | 225 | 26 |
| Day +90 | 4 | 117 | 250 | 15 |
| Day +117 | 2 | 610 | 1,555 | 333 |
| Day +131 | 3 | 928 | 2,246 | 680 |
| Day +190 | 100 | 386 | 829 | 276 |
| Day +250 | 428 | 479 | 732 | 207 |
| Day +279 | 619 | 866 | 1,621 | 538 |
| Day +445 | 785 | 561 | 488 | 180 |

Values indicate absolute peripheral blood immune cell subset counts measured by flow cytometry. Day 0 indicates stem cell infusion.
